# Correction: Development and evaluation of PlasmoPod: A cartridge-based nucleic acid amplification test for rapid malaria diagnosis and surveillance

**DOI:** 10.1371/journal.pgph.0003438

**Published:** 2024-06-25

**Authors:** Philippe Bechtold, Philipp Wagner, Salome Hosch, Michele Gregorini, Wendelin J. Stark, Jean Chrysostome Gody, Edwige Régina Kodia-Lenguetama, Marilou Sonia Pagonendji, Olivier Tresor Donfack, Wonder P. Phiri, Guillermo A. García, Christian Nsanzabana, Claudia A. Daubenberger, Tobias Schindler, Ulrich Vickos

The 12^th^ author’s name is spelled incorrectly. The correct name is: Christian Nsanzabana.
