# Supplementary figures and images for: Development and evaluation of PlasmoPod: A cartridge-based nucleic acid amplification test for rapid malaria diagnosis and surveillance
Source: PLOS Glob Public Health. 2023 Sep 27;3(9):e0001516. doi: 10.1371/journal.pgph.0001516 (PMC10529553; doi:10.1371/journal.pgph.0001516)

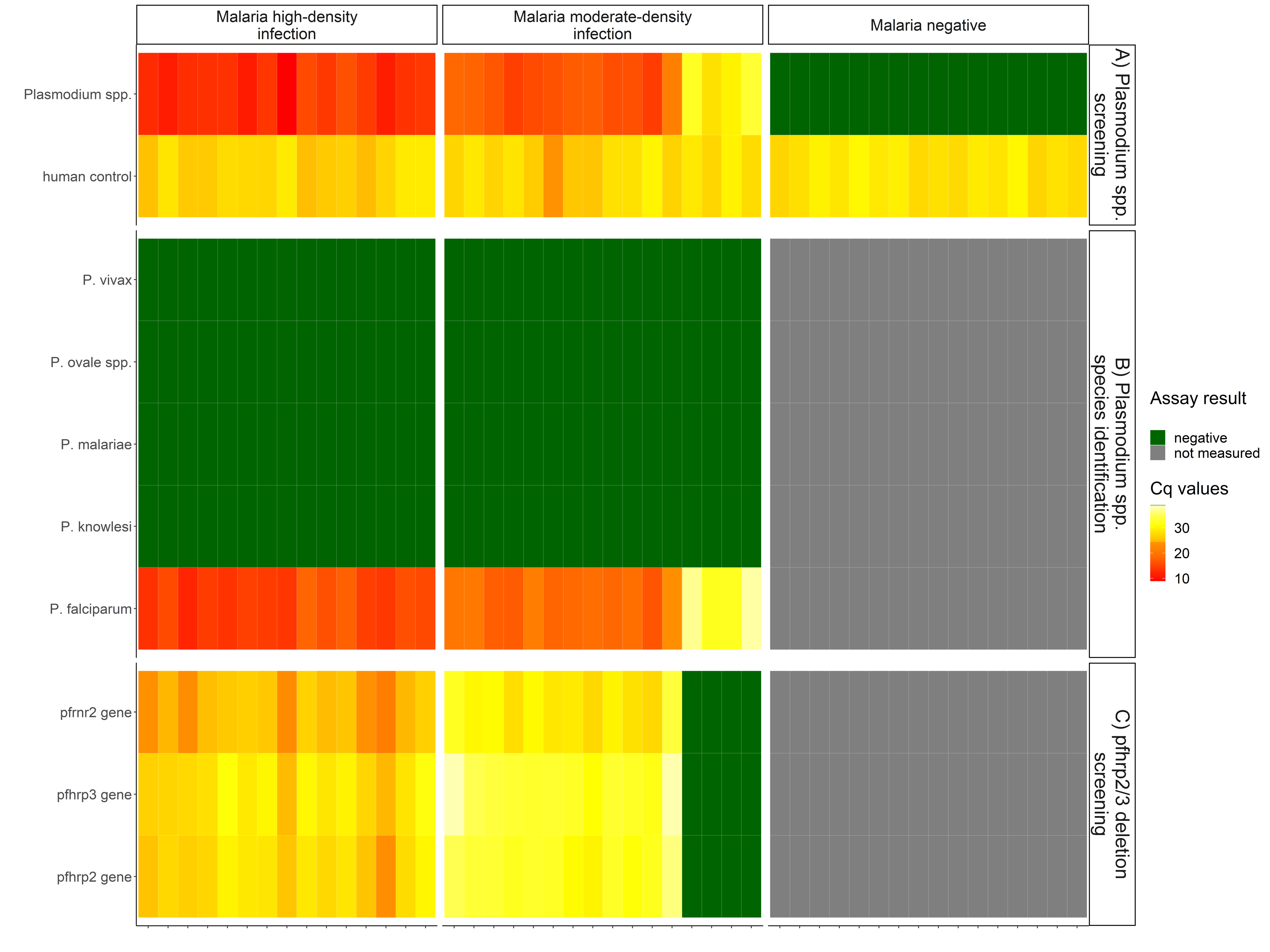

Supplement: S1 Fig — Three different molecular assays were used to (A) screen for Plasmodium spp. parasites, (B) identify Plasmodium spp. species and (C) detect pfhrp2/3 gene deletion. Each child is represented in a column stratified according to malaria infection status. Green colors represent negative measurements for the respective qPCR assay, while grey colors were chosen for tests which were not conducted. All tests were run on the Bio-Rad CXF96 qPCR instrument. (TIF) [file pgph.0001516.s001.tif]
