# Supplementary material for: Development and evaluation of PlasmoPod: A cartridge-based nucleic acid amplification test for rapid malaria diagnosis and surveillance
Source: PLOS Glob Public Health. 2023 Sep 27;3(9):e0001516. doi: 10.1371/journal.pgph.0001516 (PMC10529553; doi:10.1371/journal.pgph.0001516)
Supplement: Attachment — Submitted filename: plasmopod_response_reviewers_20230625.docx [file pgph.0001516.s003.docx]

Allschwil, May 23^rd^ 2023

**Re: Response to Comments raised by Editor and Reviewers**

1. Please send a completed 'Competing Interests' statement, including any COIs declared by your co-authors. If you have no competing interests to declare, please state "The authors have declared that no competing interests exist". Otherwise please declare all competing interests beginning with the statement "I have read the journal's policy and the authors of this manuscript have the following competing interests:"

**Answer:** We have added a Conflicts of Interest statement on page 37 of the revised manuscript version.

2. Please amend your detailed Financial Disclosure statement. This is published with the article. It must therefore be completed in full sentences and contain the exact wording you wish to be published.

a. State what role the funders took in the study. If the funders had no role in your study, please state: “The funders had no role in study design, data collection and analysis, decision to publish, or preparation of the manuscript.”

b. If any authors received a salary from any of your funders, please state which authors and which funders. If you did not receive any funding for this study, please simply state: “The authors received no specific funding for this work.”

**Answer:** We have added a Financial Disclosure statement on page 37 of the revised manuscript version.

3. Please provide separate figure files in .tif or .eps format only and remove any figures embedded in your manuscript file. Please also ensure that all files are under our size limit of 10MB.

For more information about figure files please see our guidelines:

https://journals.plos.org/globalpublichealth/s/figures

<https://journals.plos.org/globalpublichealth/s/figures#loc-file-requirement>

**Answer:** We have uploaded separate figure files and removed all figures embedded in the revised manuscript file.

5. In the online submission form, you indicated that "The data that support the findings of this study are available from the authors upon reasonable request". All PLOS journals now require all data underlying the findings described in their manuscript to be freely available to other researchers, either 1. In a public repository, 2. Within the manuscript itself, or 3. Uploaded as supplementary information.

This policy applies to all data except where public deposition would breach compliance with the protocol approved by your research ethics board. If your data cannot be made publicly available for ethical or legal reasons (e.g., public availability would compromise patient privacy), please explain your reasons by return email and your exemption request will be escalated to the editor for approval. Your exemption request will be handled independently and will not hold up the peer review process, but will need to be resolved should your manuscript be accepted for publication. One of the Editorial team will then be in touch if there are any issues.

**Answer:** We have uploaded all raw data as a supplementary data file.

**Additional Editor Comments (if provided):**

The manuscript was reviewed by three independent experts. All three reviewers identified the study results as potentially important for the malaria diagnostics field, but only with more detailed information on the methods, further description of the scope to introduce PlasmoPod into remote public health care settings, and a transparent discussion on the limitations of the study design and product. Please pay particular attention to the following requests and concerns raised by the reviewers:

1. Request for supplementary data for full transparency

**Answer:** We have uploaded all raw data as a supplementary data file.

2. Request for more information on the product including cost, throughput, and potential resourcing/supply challenges

**Answer:** We have addressed these questions and provided the requested information in different parts throughout the revised text of the manuscript.

3. Concerns about lack of testing in a malaria-endemic setting and robustness of associated claims about this potential

**Answer:** We have addressed these concerns in the Discussion section and acknowledge that further testing should be done.

4. Request for clarity on the study design, including the small sample size

**Answer:** We were able to collaborate with our colleagues from Equatorial Guinea and conducted an additional performance evaluation of PlasmoPod using a second, larger and independent dataset and therefore increased our samples size.

5. Concerns about the lack of low density infections

**Answer:** We have added an additional dataset comprising of 102 asymptomatic persons with low parasite densities from Bioko island, Equatorial Guinea.

6. Concerns about the Hb level diagnostic approach

**Answer:** Following the reviewers suggestion, we have removed this diagnostic approach.

7. Request for more detail on the scope for species-specific diagnosis

**Answer:** We have addressed the scope for species-specific diagnosis in the Discussion section.

**Reviewer #1:**

Bechtold and colleagues tested a novel, portable qPCR device for P. falciparum diagnosis. They also evaluate of a rapid DNA/RNA extraction protocol, which is equally crucial for low-resource settings. They evaluated the method on cultured parasites, and on a small set of clinical samples. While the novel method has the potential to improve malaria surveillance, several points need to be addressed before publication.

**Answer:** We would like to thank reviewer #1 for his/her work, taking these comments into consideration significantly improved the manuscript.

Main comments:

1) The information on PlasmoPod/diaxxoPCR is quite sparse. There is a miniature figure of the instrument as part of figure 1. Please add a larger figure, and also add a figure of the chip.

**Answer:** We would like to thank the reviewer for the comment on Figure 1. We agree that this figure can be improved to better explain the concept of the diaxxoPCR device and the PlasmoPod. Therefore, we have updated Figure 1. The accompanied text has also been updated (page 12, lines 232-239)

What is meant by “next generation qPCR instrument” (line 65)? How many samples can be run in parallel? Can samples and a standard curve be run on the same chip, or is this not needed?

**Answer:** For us the term "next-generation" refers to a new generation of PCR devices that offer various advantages over the standard qPCR platforms commonly found in most laboratories. These advantages can range from faster cycling programs and smaller sizes to lower costs. However, as this term may not be widely recognized, we have opted to remove it (page 4, line 74).

To clarify the sample throughput, we have added the following text: *The cartridges are equipped with a total of 20 wells that can be loaded according to specific requirements. Each well accommodates a single qPCR assay to measure one sample. With a single qPCR assay, the maximum number of patient samples per cartridge is 20. However, when incorporating controls or a standard curve, the number of samples per cartridge will be reduced accordingly* (page 4, line 80-84).

Can the same assay be run on other portable qPCR instruments, or is it limited to the diaxxoPCR (e.g. the one tested here: <https://journals.plos.org/globalpublichealth/article?id=10.1371/journal.pgph.0000454>)?

**Answer:** The PlasmoPod is based on a published qPCR assay for *Plasmodium* spp. detection. Therefore, the assay itself (based on the oligonucleotides detecting the parasite) can be run virtually on any qPCR platform. However, the speed (< 30 minutes) and the storage/shipment conditions of the reagents (room temperature) can only be achieved by implementing the qPCR assay on the cartridges compatible with the diaxxoPCR device. The types of cartridges used in this study cannot be run on other devices.

What is the cost of the instrument, and what is the per sample cost?

**Answer:** The diaxxoPCR device is sold at a price of approximately EUR 3’000 - 4’000. Due to the minimal volume of RT-qPCR reagents needed for the reaction, the costs associated with the PlasmoPod are lower compared to a standard RT-qPCR assay. Based on our estimation, the consumables and reagents costs per sample amount to approximately EUR 1.5 when produced in small-size batches. It's important to note that these costs are subject to further reduction with large-scale production of the PlasmoPod. This information has been added to the Introduction section (page 4, lines 88-91).

2) The main limitation is the small and biased dataset of only 47 field samples used to evaluate the new PCR. The lowest parasite is 54 parasites/uL, i.e. much higher than what would be observed in a real-world setting. How were these samples selected? Why were no samples with lower density included?

**Answer:** This study represents an initial investigation into the PlasmoPod approach for malaria diagnosis and surveillance. However, to establish its efficacy and reliability, additional studies must be conducted. We are committed to conducting further research directly in the settings where molecular malaria tests are most needed. Moreover, we have incorporated an additional dataset comprising 102 asymptomatic individuals from Bioko Island who tested positive for malaria. We recognize the significance of including low-density infections from asymptomatic individuals to comprehensively evaluate this new technology. However, when it comes to malaria, we believe that samples collected in a paediatric hospital in Bangui are considered “real-world”. In the Discussion section, we have also addressed the limitations pertaining to the sample set used for validation, emphasizing the need for further testing (page 31, lines 536-539).

Overall, our study lays the foundation for future investigations and underscores the necessity of extensive validation and ongoing research in order to establish the effectiveness and utility of the PlasmoPod approach in the field of malaria diagnosis and surveillance.

3) Following the point above, the 95% detection probability based on a probit analysis of the field samples is included. Such an analysis requires a large number of samples around the LOD of each method. Given the very limited number of samples available, the results are unreliable and completely implausible. For example, the 95% detection probability for HRP2-based is calculated as 11,000 parasites/uL. This is approx. 100-fold higher than other studies that tested HRP2-based RDTs (as correctly stated in line 56). While differences are likely due to different RDTs used, study population characteristics (i.e. different mean parasite densities), and technical variation of density calculation based on qPCR, such large differences are implausible.

Unless the authors are able to run many more samples (in particular low density samples), this analysis cannot be conducted.

**Answer:** We agree that the graphical representation is not appropriate for the dataset presented here. We have therefore decided to remove this figure.

4) The authors divide their samples in high density (>5000 parasites/uL) and low density (<5000 parasites/uL). This seems like an odd choice. RDTs now reach limits of detection of <100 parasites/uL. The samples included in the low density groups are in no way low density with respect of diagnosis. Almost all of them will easily be detected by RDT. These groups should be combined, and references to low-density samples removed, as there are almost none.

**Answer:** As a parasitological endpoint in clinical studies a cut-off of 5000 parasites/uL is often used to define symptomatic and sometimes severe malaria. Therefore, we think using this cut-off to divide the clinical cohort is appropriate. However, we agree that the term “low density” might be misleading, we have changed the term for the < 5000 parasites/uL category to “moderate parasite density group”. For clarification we have added additional text and three references citing studies using this cut-off in the Materials section (page 9, lines 182-184). This change is also reflected in Figures 3, S1 and in Table 2.

5) Lines 190-205: Was the cultured blood spotted on DBS, and a single 3mm punch used for extraction? If so, it is difficult to understand how a positive result can be obtained at 0.02 parasites/uL. One DBS punch is expected to correspond to 1-2 uL of blood (line 353). Assuming a single parasite was present in 2 uL, the theoretical lowest LOD will be 0.5 parasites/uL.

If for this test DNA+RNA was extracted from whole blood, please add this info. It might also be worth do add sentence or two to the discussion explaining the importance of this difference (lines 401-402)

The situation would of course be different if the target RNA was secreted to the blood. If this is the case, please describe.

**Answer:** We would like to thank the reviewer for this suggestion. To clarify that we analysed total nucleic acids (DNA and RNA) we modified the text in the Results section (page 14, line 274) and in the Discussion section (page 32, lines 572-575)

6) The authors develop a threshold based on hemoglobin levels as diagnostic marker for malaria and compare the new PCR against it. While the relationship between low Hb levels and infection have long been established, it is well known that the correlation is at best moderate. It is very odd to compare a new test to an approach that was developed ad hoc based on a very small dataset, is not used by control programs, and not recommended by the WHO. This analysis should be removed.

**Answer:** We would like to thank the reviewer for this insightful feedback. Upon careful consideration of the argumentation presented, we have made the decision to remove the additional analysis entirely from the manuscript. We acknowledge the validity of the reasoning behind this suggestion and believe that this adjustment will strengthen the overall focus and clarity of our work.

7) For each sample (cultured and clinical), all data including Cq value by conventional and PlasmoPod qPCR, parasite density by microscopy (for clinical samples), and RDT results (for clinical samples), should be included as a supplementary file. The clinical data can be presented complete de-identified. There is no reason this data is only available from the authors upon reasonable request. This is of particular importance as several co-authors are employed by the company producing the PlasmoPod and thus have a commercial interest.

**Answer:** We agree with this statement and have added all raw data as a supplement.

8) I commend the authors for keeping their discussion short and to the point!

**Answer:** Thanks for the feedback.

Minor comments:

Lines 59-62: Re-phrase this sentence, as it implies that well-equipped centralized laboratories are only available in in resource-constraint settings.

**Answer:** We have rephrased the sentence (page 3, line 68-70).

Line 68: What is meant by ‘Control strategy’? Should that read amplification strategy?

**Answer:** We have added the word “temperature” for clarification (page 4, line 77)

Lines 74-76: Please provide a reference

**Answer:** Thanks for the suggestion. We have provided the reference.

While the technology has the potential for decentralized diagnosis, for the current study all samples were tested at the Swiss Tropical and Public Health Institute. Thus, the term ‘decentralized’ should not be included in the title.

**Answer:** Thanks for the feedback. The term “decentralized” has been removed from the title.

Line 152: Typo: diaxx-oPCR

**Answer:** Thanks for the spotting the typo. We have corrected the typo.

Figure 4: For added clarify, please add what green and grey means to the little plot at the right showing the color coding of the Cq values.

Also, the figure might be easier to read if the height of the boxes was reduced.

**Answer:** Thanks for the feedback. Figure 4 has been updated according to the suggestion and has moved to the supplementary data (it is now Figure S1)

**Reviewer #2:**

Bechtold et al., describe PlasmoPod, a cartridge-based nucleic acid (NA) amplification test ran on diaxxoPCR platform for the detection of Plasmodium spp. NA is rapidly extracted from DBS, and the PCR is completed in less than 30 minutes. The performance of this device is compared to commercially and well-established molecular detection platforms with established extraction and NA amplification and detection methods. The diaxxoPCR platform has recently been described in the development of other cartridge-based NA amplification tests for other infectious diseases including the detection of SARS-CoV-2. The authors use previously published Plasmodium primer sets to create PlasmoPod. In this study, the authors describe the analytical performance of the PlasmoPod using Plasmodium NA obtained from cultures, the WHO International Standard for P. falciparum DNA for NAAT-based assays, and field collected samples. The designed, execution and the analysis of the study is well thought through, and some of the analyses performed are important in assessing the performance of the PlasmoPod device. I have some comments for the authors.

**Answer:** We would like to thank reviewer #2 for his/her work and by taking his/her comments into consideration the manuscript improved considerably.

MAJOR Comments

1. The PlasmoPod and diaxxoPCR were used to evaluate a previously developed single target malaria PCR. The test was performed in a highly controlled environment, probably by the platform and/or test developers. The authors cannot claim that this test/platform is suited for deployment to satellite public health laboratories or remote health care settings since they had an opportunity to test the performance of the platform in the field settings in CAR, but they choose to bring the specimen back to Switzerland. Until they deploy the test/platform in the field settings where they think the platform is suited, then right now all they can do is speculate. The test/platform can analyze NA from arguably a straight forward, and simple protocol. However, in clinical labs and other low or limited resource (including manpower) setting, sample extraction process is a limiting factor when compared to a platform such as the GeneXpert, a cartridge-based platform which requires less than 2 minutes hand-on sample loading and includes sample extraction. It is good to remember that in a clinical lab setting, many different tests are run at the same time by many technicians, and that’s why what might seem straight forward when developing a test in a highly controlled environment might produce different outcome in high throughput or resource limited settings.

**Answer:** Thanks for this important comment and in general we agree with the reviewer on this topic. In this manuscript we describe our PlasmoPod approach for malaria diagnosis and surveillance for the first time. We are fully aware that more validation test need to be done. Further studies are planned and they will be conducted directly in the settings where molecular malaria tests are needed most. A paragraph about limitations regarding the sample set for validation and that further testing is required has been added to the Discussion (page 31, lines 536-539).

While the GeneXpert system offers numerous advantages, one limitation of the GeneXpert system is its lack of a malaria test in its portfolio, which restricts its utility in addressing the global burden of malaria.

2. Using hemoglobin value as a standalone malaria diagnostic parameter has no clinical relevance and is absolutely misleading since hematological parameters can be impacted by many factors including other infectious diseases, clinical or genetical conditions. However, hemoglobin and platelet can be important parameters in understanding severity of malaria disease, and along with other specific diagnostic methodologies for the detection of malaria parasite such as those described here (molecular, antigen detection), hematological parameters (hemoglobin and platelet) can provide an additional layer of information or algorithm that can be used to increase diagnostic test specificity. The authors must revisiting/rewriting and reanalyzing hemoglobin as a diagnostic parameter since it cannot be used for malaria diagnosis as a standalone diagnostic tool, but as a tool that can complement other methods.

**Answer:** We would like to thank the reviewer for this insightful feedback. We are convinced by this argumentation and have decided to remove this additional analysis completely from the manuscript

Other comments

1. There are other malaria tests available as analyte specific reagent (ASR) and can be available for most PCR platforms already in the market such as this one https://www.ncbi.nlm.nih.gov/pmc/articles/PMC4026594/. The authors should discuss the performance of their test/platform and compare the performance to other platforms in the market.

**Answer:** Thanks for the suggestion. We have improved the Discussion section and acknowledge the considerable number of molecular malaria tests that have been published during the past decade (page 33, lines 593-594).

2. The PlasmoPod has only a single target, Plasmodium at genus level. Malaria epidemiology is shifting due to elimination efforts and climate chance, and places such as Ethiopia and Somalia have two different malaria species circulate that require different treatment options. The authors must address this as one of the limitations of this test and how do they envision overcoming this without making testing increasingly complicated and expensive.

**Answer:** Currently, the PlasmoPod is designed to detect conserved NA sequences present in all human pathogenic *Plasmodium* spp. species. However, future development of this platform could focus on the design and validation of cartridges specifically designed for identifying individual *Plasmodium* spp. species. We have added additional text on *Plasmodium* species identification to the Discussion section (page 33, lines 598-603).

3. Not all diagnostics tests are perfect, but there must be a balance such as the ease of use, reagent and platform cost, the need for infrastructure etc. PlasmoPod sensitivity is same as that of PfHRP2-based RDT. The authors have not addressed reagent and platform cost. This is important, one of the main reasons why molecular tests are still not widely deployed for malaria testing. For marginal increase in sensitivity, the authors should present strong argument why this test should be considered including low cost for the reagents and platform (if this is true; must compare cost to current platforms in the market of the same class/caliber), and potential for low maintenance etc. The authors must remember GeneXpert has penetrated the low resource settings mostly because of the TB program, and the infrastructure including the supply chain. How do the authors see such a platform penetrating the market including having readily available supply chain?

**Answer:** The diaxxoPCR device, being both CE marked and IVD-D certified, is readily available for commercial purchase at a list price ranging from EUR 3000 to 4000. Compared to the cost of a GeneXpert system, this represents a fraction of the price. Additionally, the use of low volumes of RT-qPCR reagents in the reaction contributes to lower overall costs compared to a standard RT-qPCR assay. In our estimation, the consumables and reagents costs per sample amount to approximately EUR 1.5 when produced in small-size batches. Furthermore, scaling up the production of PlasmoPods would likely lead to a further reduction in costs. It is important to note, however, that these cost estimates do not include additional expenses associated with regulatory compliance and distribution. Since the PlasmoPod is not yet a commercially available product, it is challenging to provide a precise estimate for these particular costs.

At the current early stages of product development and evaluation, there are presently no plans in place for large-scale development, maintenance, or establishment of supply chains for the PlasmoPod. Nevertheless, one of the major advantages of the diaxxoPCR device is its utilization of qPCR technology, which allows for straightforward adaptation of existing qPCR assays to the platform.

I have a personal appreciation for the GeneXpert system and the commendable impact it has had on global diagnostics for tuberculosis. Considering its wide market presence in malaria endemic regions, it seems logical that they would offer a malaria test. However, to the best of my knowledge, such a test is currently not available. It is important to recognize that even with extensive market penetration, the effectiveness is limited if the tests are not readily accessible. This was evident during the initial stages of the COVID-19 pandemic, when GeneXpert SARS-CoV-2 tests were scarce outside of the United States. Consequently, relying solely on a single platform could pose risks to public health systems, especially if tests or diagnostic platforms could be affected by export restrictions.

4. The authors have not addressed any study limitations including the study design and the results obtained.

**Answer:** We would like to thank the reviewer for raising this point. Throughout the entire Discussion we have added paragraphs about current limitations (page 31, lines 536-541).

5. The authors describe three different PCR methods, with limited or scanty details, mostly referencing previously published methods. They also have used different specimen types (e.g., DBS extracted using different protocols etc.). It is a little hard to follow as they describe these tests back and forth but what is even more frustrating for example, starting lines 346-361, they attempt to describe how PlasmoPod performs equally as good as the other PCR methods, using details in the method that we either referenced or not chronologically well presented.

**Answer:** Thank you for your comment. We acknowledge that the previous paragraph may have lacked clarity. To address this, we have rewritten it to provide a more concise and focused statement.

**Reviewer #3:**

This manuscript reports the evaluation of a newly developed molecular diagnostic for malaria infection. It includes an assessment of the LOD by using a serial dilution of a cultured P. falciparum sample; tests for cross-reactivity with other commonly circulating pathogens; and finally a performance evaluation with samples from febrile patients seeking care at a hospital in Central African Republic. Diagnostic performance is compared to two standard and high-sensitivity molecular PCR-based assays. The diagnostic performance of the new PlasmoPod assay looks very impressive and encouraging. Some discussion of the next steps and further work needed to confirm these results and further evaluate the assay would be important to include, e.g. in the field across diverse patient groups and malaria species.

**Answer:** We highly appreciate the encouraging comments from Reviewer #3. We fully agree that further validation tests are needed. In this manuscript we describe our PlasmoPod approach for malaria diagnosis and surveillance for the first time. Further studies are planned and they will be conducted directly in the settings where molecular malaria tests are needed most. A paragraph about limitations regarding the sample set for validation and that further testing is required has been added to the Discussion (page 31, lines 536-539).

The assay is currently not species-specific. Any scope to address this should be described in the paper.

**Answer:** Currently, the PlasmoPod is designed to detect conserved NA sequences present in all human pathogenic Plasmodium spp. species. However, future development of this platform should focus on the design and validation of cartridges specifically designed for identifying individual Plasmodium spp. species. We have added additional text on Plasmodium species identification to the Discussion section (page 33, lines 598-603).

More description of diaxxoPCR device itself and the PlasmoPod cartridges would be useful to give an idea of how this tech could feasibly be used: e.g. cartridge storage restrictions, fragility (how feasible to transport in back of a truck?), size and weight, indicative cost range, throughput (samples per hour).

**Answer:** Thanks for the comment. We have added more information about the device and the PlasmoPod cartridges, including better illustrations and pictures (Figure 1) Additional text providing more details about the technology can be found in the Introduction section (page 4, lines 80-84) and Result section (page 12, lines 232-239).

The “Hb-8.8” method is not standard practice. I would strongly recommend excluding from this evaluation; it does not add anything.

**Answer:** We would like to thank the reviewer for this insightful feedback. This issue was raised by all three reviewers and therefore we have decided to remove this additional analysis completely from the manuscript.

Introduction:

- Line 52: 419m RDTs sold in 2020 was the estimate of PQ-approved assays only, could add that detail

**Answer:** Thanks for this information. We have added it to the Introduction (page 3, line 57)

- Line 54: Reference 7 dates to 2006 and RDTs have improved in quality since then. A more recent evaluation of RDT performance to check: <https://apps.who.int/iris/bitstream/handle/10665/276193/9789241514958-eng.pdf?ua=1>

**Answer:** Thanks for the link to the program. It’s very impressive and important work which we have added to the Introduction (page 3, lines 58-60). The quality of the RDTs has improved. The sensitivity however remains limited as they detect antigens without involving target molecule amplification.

- Line 71: car battery: can other types of batteries also be used? Are these included as part of the kit? Can the assay also be connected to the mains power supply?

**Answer:** The device can be run from a standard electrical power supply (220V), but for mobile testing applications the device can be run from a low voltage electrical source such as a car battery or solar-powered battery. For clarification we have modified the sentence (page 4, line 85).

- Mention how conserved the 18S ribosomal DNA and RNA are: would these be expressed by zoonotic species as well as all 5 human Plasmodia?

**Answer:** Yes, the target sequence is expressed by all 5 human *Plasmodium* spp. species. To demonstrate this, we have successfully measured clinical samples positive for *P. vivax*, *P. ovale* spp. and *P. malariae*. The results have been added to Figure 2 and the text in the Results section was modified accordingly (page 14, lines 275-277).

Methods:

- Suggest moving Figure 1 into Methods section.

Answer: We would like to thank the reviewer 3 for the comment on figure 1. Since there was also another comment by reviewer 1, we decided to improve Figure 1 for better demonstration of the diaxxoPCR device and the PlasmoPod. Therefore, we have updated Figure 1. The accompanied text has also been updated (page 12, lines 232-236)

- Line 88: “admitted to the emergency department”. How were patients selected for inclusion?

**Answer:** We have added more information about the recruitment process (page 7, lines 142-143)

- Contradiction in the RDT brand used in the study: CareStart (line 90) or A&B Professional (line 463). Which was used? Note that CareStart was issued a WHO Notice of Concern in 2021; A&B Professional has not yet received WHO Pre-qualification for their assays. Any future evaluations should prioritise WHO PQ-approved RDTs if possible. Could refer to this limitation in the Discussion.

**Answer:** Thanks a lot for pointing this out. We used the Pf/Pan mRDT from A&B Professional. The CareStart RDT was not used in this study. We apologize for any confusion caused by this. We have corrected it in the Materials section and added one sentence that using a RDT without WHO PQ is a limitation to the study into the Discussion section.

- Line 91: given the results with microscopy, worth stating the experience of the microscopists reading the slides. Were slides double-read?

**Answer:** Yes, the sildes were double-read.

- Need to add target sample size and justification for this.

**Answer:** We added samples collected from an additional 102 individuals to increase the samples size. However, while the initial evaluation of PlasmoPod using samples from 149 individuals shows promise, further validation studies are necessary to fully assess its performance and reliability. This statement has also been added to the Discussion section.

- Line 102: write in full “NEM”

**Answer:** We have added the full name (New Extraction Method) of the extraction protocol (page 8, line 156)

- Line 124: the cut-off for high/low parasitaemia of 5000p/ul seems arbitrary and convenient given the samples collected. Justify this threshold, especially if the assay’s main strength is detection of very low density infections. A cut-off of 2000p/ul would have been consistent with the upper threshold from the WHO RDT evaluation protocols.

**Answer:** Thanks for the suggestion. As a parasitological endpoint in clinical studies a cut-off of 5000 parasites/uL is often used to define symptomatic and sometimes severe malaria. Therefore, we think using this cut-off to divide the clinical cohort is appropriate. For clarification we have added additional text and three references citing studies using this cut-off in the Materials section (page 9, lines 182-183). This change is also reflected in Figures 3, S1 and in Table 2.

- Line 132: “amplification and detection of Plasmodium spp”: possible to give more detail on the type of technological approach that’s used?

**Answer:** We added the following text to the Material section: “*The Plasmodium spp. assay used for PlasmoPod is a TaqMan probe-based qPCR assay which uses a 6-Carboxyfluorescein (6-FAM) labelled probe to enable the detection of a specific amplification product produced during PCR.”* (page 6, lines 105-107)

- Line 149: how is the heating of the DBS in Chelex to 95C done in the field? A water bath would be needed?

**Answer:** A standard laboratory heat block was used, a water bath would also work. For clarification we have added a photo of our simplified laboratory setup which includes all equipment required to run PlasmoPods (Figure 1C).

Results:

- Fig 3B: could the y-axis scale be absolute numbers of samples?

Answer: Thanks for pointing out the missing y-axis description. We have now added the description.

- Fig 4: mention pfmr2 in the legend; what Cq value was used as the cut-off for the green?

**Answer:** Thanks for the feedback. The Cq value is assay specific and cut-offs of Cq 40 was chosen. Figure 4 has been updated according to the suggestion and has moved to the supplementary data (it is now Figure S1)

- Line 290: specify P. falciparum in the header here: no other species was evaluated

**Answer:** Thanks for the suggestion. After rearranging this part of the Result, we removed the title.

- Lines 301 and 305: would compare like-with-like: quote figures from the same sample groups

**Answer:** Thanks for the suggestion, we have re-written this section in order to make it more concise (page 23, lines 416-419)

- Table 2: add in absolute numbers

**Answer:** The absolute number have been added to Table 2

- Fig 5: Interesting figure/analysis. Possible to explain the modelled >50% detection probability of the HRP2 RDT at such low densities? The specificity doesn’t seem to explain completely.

**Answer:** We agree, in general the parasite densities were too high for such as graphical representation. Therefore, and in line with a comment from Reviewer #1 we decided to remove this analysis.

- Lines 353-354: suggest moving this sentence to end of paragraph (though actually would fit into Discussion section better)

**Answer:** Thanks for the excellent suggestion, we have moved this statement to the Discussion section.

- Line 361: claim about cost-effectiveness needs a basis.

**Answer:** We removed the term cost-efficient from the Results section and added more detailed information about the costs in the Introduction section:

“Due to the minimal volume of RT-qPCR reagents needed for the reaction, the costs associated with the PlasmoPod are lower compared to a standard RT-qPCR assay. Based on our estimation, the consumables and reagents costs per sample amount to approximately EUR 1.5 when produced in small-size batches. It's important to note that these costs are subject to further reduction with large-scale production of the PlasmoPod.”

- Fig 6: add unit (Cq) to y-axis title; put p-value in simpler format (e.g. <0.001)

**Answer:** Thanks for the suggestion, we have updated the figure accordingly (now labelled as Figure 5)

Discussion:

- Line 401: “indicates”; the LOD was determined with a small number of replicates, recommend “suggests”?

**Answer:** Agree. We changed the wording (page 32, line 556)

- Line 409: “lower parasite densities” would instead say “<5000 p/ul”.

**Answer:** We agree that the term “low parasite density” might be misleading, therefore we have changed the term for the < 5000 parasites/uL category to “moderate parasite density group” throughout the entire manuscript.

- This initial evaluation gives very encouraging results, but it would be important to discuss the limits of the evaluation and the next steps needed to further understand the assay performance, especially for its target applications.

**Answer:** We fully agree that further validation is needed. In this manuscript we describe our PlasmoPod approach for malaria diagnosis and surveillance for the first time. Further studies are planned and they will be conducted directly in the settings where molecular malaria tests are needed most. A paragraph about limitations regarding the sample set for validation and that further testing is required has been added to the Discussion (page 31, lines 536-541).

- Also important to discuss the assay limitations: PlasmoPod detects Plasmodium NA, but does not differentiate species. What scope to develop a species-specific assay? Are there similar high-copy number potential markers that could be used?

**Answer:** Yes, future development of this platform should focus on the design and validation of cartridges specifically designed for identifying individual Plasmodium spp. species. We have added additional text on Plasmodium species identification to the Discussion section (page 32, lines 578-583).

And any scope for an application to screen for drug resistance markers, even if this had a lower sensitivity, parasitaemia in patients would usually be higher than what’s required for detection of asymptomatic infections.

**Answer:** We appreciate this comment and we also believe that adding cartridges for characterization of anti-malaria drug resistance markers could be particularly valuable in supporting malaria surveillance efforts. We have added a paragraph on future direction of developments (page 33, lines 598-601).

- PlasmoPod is described as “Cost-effective” a few times. Evidence of this in the context of a specific application is needed to support this statement.

- Giving a target cost range for the test would also be very useful; what does “low cost” mean (line 393)

**Answer:** We added more detailed information about the costs in the Introduction section (page 4, lines 88-91)

- LAMP is another portable molecular diagnostic: some reference and comparison should be made to this.

**Answer:** Thanks for the suggestion. We have improved the Discussion section and acknowledge the considerable number of molecular malaria tests and isothermal amplification methods such as LAMP and RPA, which have been published in the past decade (page 33, lines 593-594). A direct comparison between LAMP and PlasmoPod would be an interesting approach for a field study in a malaria endemic region under real-world conditions.
