# Supplementary material for: Development and evaluation of PlasmoPod: A cartridge-based nucleic acid amplification test for rapid malaria diagnosis and surveillance
Source: PLOS Glob Public Health. 2023 Sep 27;3(9):e0001516. doi: 10.1371/journal.pgph.0001516 (PMC10529553; doi:10.1371/journal.pgph.0001516)
Supplement: Attachment — Submitted filename: plasmopod_response_reviewers_rev02.docx [file pgph.0001516.s004.docx]

Allschwil, August 30^th^ 2023

**Re: Response to Comments raised by Reviewers**

Reviewer #1: The authors have addressed my comments well. My main remaining major comment is that sometimes the structure of the manuscript makes it a bit hard to follow the main findings. Several different types of sample (culture, archived RDTs, DBS), study populations (clinical, asymptomatic), and extraction protocols were studied, it sometimes is hard to grasp what exactly was done. Some suggestions for improvement:

**Answer:** We sincerely appreciate the time and effort spent by Reviewer #1 to carefully assess our manuscript. The comments raised by the Reviewer have significantly improved the manuscript.

Lines 240-259: The previous paragraph describes the simplified Chelex extraction, but I believe this analysis was done on DNA/RNA extracted using another method. It would help to clarify that this analysis does not include the impact of the extraction protocol used.

**Answer:** Thanks for the comment. The primary objective of the analytical performance evaluation was to assess the performance of the qPCR itself. Thus, we utilized purified NAs, without considering the rapid extraction procedure's potential impact on the evaluation. In order to provide clarity, we have included this sentence in the Results section (page 12, lines 242-244).

Lines 267-268: It could help to replace ‘samples’ with ‘archived RDTs’ in the title to highlight that another sample type than elsewhere was used. Likewise, the title on lines 298-299 could include the term ‘DBS’. It would also be good to repeat that different extraction methods were used for RDTs and DBS, which likely has an impact on sensitivity.

**Answer:** Thanks for the comment. We have modified the manuscript accordingly, including the title on page 18, lines 357-358. We also added a short paragraph to the Discussion section (page 24, lines 484-488).

Lines 269-276: It might be good to repeat here that these 102 samples were selected based on a previous PCR screening. Else the reader might be confused on why all of them are positive.

**Answer:** We have introduced an additional sentence to clarify the sample selection (page 13, lines 274-275).

Lines 298-374: Given the clear aim of this study is to evaluate the PlasmoPod, I find it quite confusing that after the data obtained by the RT-qPCR on the BioRad instrument is presented, a very long paragraph describes demographic patterns and data on RBCs, Hb etc, all of which is not directly relevant for the main aim of the paper. The PlasmoPod results are only given in a subsequent paragraph. Shouldn’t the BioRad vs. PlasmoPod comparison be given first, with the secondary results presented later? (RBC, Hb and similar data could even be put in a supplementary file).

**Answer:** We appreciate the reviewer's comment. However, we are of the opinion that including this additional clinical data on the symptomatic cohort is essential for a more comprehensive characterization of the context within which PlasmoPods are being evaluated.

Throughout the manuscript, the use of the terms PlasmoPod, diaxxoPCR, and Pspp18S RT-qPCR are used interchangeably which makes it harder to follow the text. For example, lines 357-361:

“During the clinical evaluation stage, PlasmoPod was run with NAs extracted by the rapid Chelex-based procedure on the diaxxoPCR instrument. As the gold standard for qualitative comparisons, we used the outcome of the highly sensitive Pspp18S RT-qPCR assay based on amplification of NAs extracted with the NEM protocol and run on the Bio-Rad CXF96 qPCR instrument.” From this text, it is not immediately clear that the Pspp18S was used on both platforms. The text could be clarified by either stating in the methods that the Pspp18S assay was always used except when a single copy assay was used for quantification, and then not repeat ‘Pspp18S’, or it should always be used.

Minor comments:

Typo on line 63: P. falciparum and pfhrp2 not in italics

**Answer:** Thanks for the comment. We have corrected this.

Typo on lines 85-86: diaxx-oPCR

**Answer:** Thanks for the comment. We have corrected this.

The Pspp18S qPCR assay is first mentioned on line 107, but only on lines 131-132 it is stated that “18S ribosomal DNA and RNA molecules were targeted [18,21] and detected by

a highly-sensitive RT-qPCR (herein referred to Pspp18S RT-qPCR assay) [19]”. It would be more appropriate to include this information when the assay is mentioned first.

**Answer:** Thanks for the comment. We have corrected this.

My comment on the LOD depending on the amount if input material might not have been fully clear. Line 448 states an LOD of 0.02 parasites/uL. This is correct when NAs are extracted from whole blood. Yet, given this study included many different sample types, it’s a bit misleading. When 5 uL blood are put on an RDT and then NAs are extracted, the assay can only be positive if at least one parasite was present. Thus, the mathematically possible lowest LOD for this assay is 0.2 parasites/uL (1 parasite in 5 uL blood). The sentence on line 448 should be rephrased to include that this LOD can be achieved for whole blood, but will be higher for RDTs and DBS.

**Answer:** Thanks for the comment. We have modified the sentence (page 23, line 454). We also added a short paragraph to the Discussion section (page 24, lines 484-488).

Line 519: No diagnosis is needed for MDA. This sentence is confusing, I don’t think MDA should be mentioned here.

**Answer:** Thanks for the comment. We have modified the sentence (page 21, line 415)

Reviewer #2: No further comments, thank you for adequately addressing all the concerns.

**Answer:** We would like to thank Reviewer #2 for the generous investment of their time and effort in thoroughly reviewing our manuscript.
